# Long-term health outcomes and risk factors for low self-rated health in non-hospitalised adults with post-COVID-19 condition: a 2.5-year cohort study

**DOI:** 10.1186/s12889-026-26532-z

**Published:** 2026-02-03

**Authors:** Anna Törnberg, Anna Svensson-Raskh, Elisabeth Rydwik, Annie Svensson, Mikael Björnsson, Daniel E. Loewenstein, Michael Runold, Judith Bruchfeld, Malin Nygren-Bonnier

**Affiliations:** 1 Division of Physiotherapy, Department of Neurobiology, Care Sciences and Society, Karolinska Institutet, Huddinge, SE-141 83 Sweden; 2 Medical Unit Allied Health Professionals, Theme Women’s Health and Allied Health Professionals, Karolinska University Hospital, Stockholm, SE-171 76 Sweden; 3 Department of Medicine Solna, Karolinska Institutet, Stockholm, SE-171 76 Sweden; 4 Department of Molecular Medicine and Surgery, Karolinska Institutet, Stockholm, SE-171 76 Sweden; 5 Department of Clinical Physiology, Theme Heart, Vascular and Neuro, Karolinska University Hospital, SE-171 76 Stockholm, Sweden; 6 Department of Respiratory Medicine and Allergy, Theme Inflammation and Aging, Karolinska University Hospital, Stockholm, SE-171 76 Sweden; 7 Department of Infectious Diseases, Theme Emergency and Reparative Medicine, Karolinska University Hospital, Stockholm, SE-171 76 Sweden

**Keywords:** Health-related quality of life, Mental health, Physical activity, Physical fitness, Post-acute COVID-19 syndrome, Recovery of function

## Abstract

**Background:**

Knowledge regarding the clinical course and prognosis in non-hospitalised individuals with post-COVID-19 condition (PCC) remains limited. This study aimed to explore the impact of PCC on physical function, physical activity, and mental health in non-hospitalised adults, and to identify risk factors for low self-rated health. Extended knowledge may inform follow-up strategies and targeted interventions in non-hospitalised individuals with PCC.

**Methods:**

A cohort study was conducted at a specialised post-COVID clinic, with assessments of physical function (six-minute walk test, one-minute sit-to-stand test, maximal inspiratory pressure, mMRC dyspnoea), physical activity (Frändin/Grimby activity scale), mental health (depression: PHQ-9; anxiety: GAD-7), and self-rated health (EQ VAS) at 12 and 30 months after COVID-19. A total of 130 non-hospitalised adults with PCC were included. Data were collected between August 2020 and December 2024.

**Results:**

Participants were predominantly middle-aged, previously physically active women. Physical and mental impairments, and low physical activity remained prevalent at follow-up, despite some improvements over time. Impaired performance in the one-minute sit-to-stand test, a Frändin/Grimby activity level < 3, and a PHQ-9 score ≥ 10 at baseline were associated with lower EQ VAS scores at follow-up.

**Conclusion:**

There were long-term negative impacts of PCC on health outcomes 2.5 years after COVID-19 in non-hospitalised individuals, including impairments in physical and mental health, low physical activity, and low self-rated health. Impaired physical function, low physical activity, and depressive symptoms were identified as risk factors for low self-rated health. These findings expand current knowledge of prognosis in PCC, underscore the need for systematic follow-up using simple clinical tools to identify individuals at high risk, and inform targeted interventions to improve long-term outcomes.

**Supplementary Information:**

The online version contains supplementary material available at 10.1186/s12889-026-26532-z.

## Introduction

Post-COVID-19 condition (PCC) is an umbrella term that encompasses the persistent and heterogeneous symptoms and impairments affecting 2–7% of individuals after initial COVID-19 disease, potentially impacting hundreds of millions globally [1, 2]. Though PCC severity varies, about 20% report severe symptoms [2]. Most individuals currently living with PCC were not initially hospitalised [2]. Being bedridden for a week or longer, female sex, being unvaccinated, recurrent COVID-19 infections, and pre-existing comorbidities are factors that increase the risk of developing PCC [3]. Individuals with PCC often experience several multisystem symptoms such as fatigue, dyspnoea, palpitations, musculoskeletal pain, and depressive symptoms which may negatively impact physical and mental functioning, physical activity, health-related quality of life, self-rated health and work ability [2–7]. There is currently no curative treatment for PCC, but tailored rehabilitative interventions show promising results [2].

Several studies of recovery following COVID-19 in previously hospitalised individuals show improvements over time, but with substantial impairments lasting up to three years after initial COVID-19 [6, 8–11]. Currently, there is a lack of data on health outcomes in non-hospitalised individuals with PCC, especially from long-term follow-ups. Previous studies in non-hospitalised individuals primarily report on patient-reported outcome measures and symptoms. While some studies in non-hospitalised individuals report impairments in physical function using objective measures, follow-up is often limited to one year [6, 12]. Hence, in non-hospitalised individuals with PCC, there is a need for long-term follow up of health outcomes more than one year after initial COVID-19 infection.

The aim of this study was twofold. First, it sought to explore the impact of PCC on physical function, physical activity, mental health, and self-rated health in non-hospitalised adults more than a year after the initial COVID-19 infection. Second, it aimed to identify risk factors for lower self-rated health. By further exploring the clinical course and prognosis of PCC in non-hospitalised individuals and identifying those at risk of poorer long-term health outcomes this study seeks to inform the development of tailored rehabilitative interventions.

## Methods

### Design, setting and participants

This prospective, observational cohort study is part of the ReCOV research project (Recovery and rehabilitation during and after COVID-19), for which a study protocol has been published elsewhere [13]. One of the overarching aims of ReCOV is to evaluate recovery and its predictors across multiple health domains following COVID-19. Participants in ReCOV are primarily recruited from the specialised, interprofessional post-COVID outpatient clinic at Karolinska University Hospital, Stockholm, Sweden, where clinical assessments are performed in adult individuals, either referred following hospitalisation due to COVID-19 or non‑hospitalised individuals with severe PCC referred from primary care or self-referred. Referral criteria for non-hospitalised individuals include the World Health Organization’s (WHO) definition of PCC, as well as severe functional impairments resulting in at least 50% disability or sick leave [14].

**Fig. 1:**
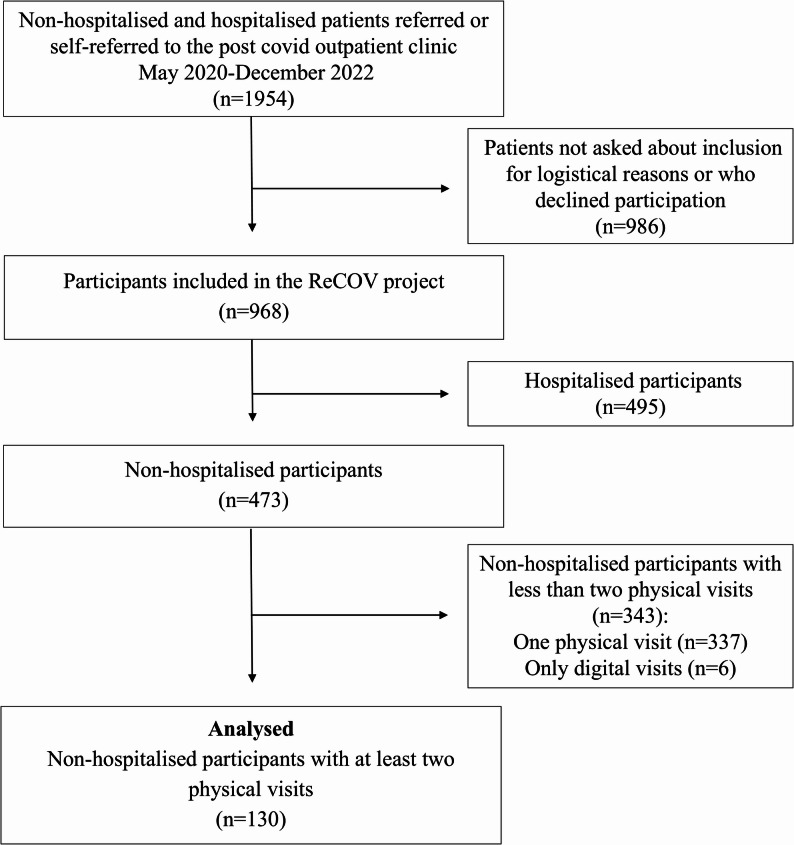
Flowchart of participants included in this study

All patients attending the post-COVID clinic were eligible for inclusion and were invited to participate in the ReCOV project. In practice, patients were invited to participate during their clinical visit; however, as detailed in Fig. 1, a proportion were not approached for inclusion due to logistical reasons (e.g., staffing constraints and variations in recruitment procedures), while others declined participation. Baseline data for both hospitalised and non‑hospitalised ReCOV participants have been reported in a separate publication, providing an overview of the full cohort [15].

While ReCOV includes both hospitalised and non-hospitalised individuals, the present study focuses specifically on non‑hospitalised individuals with PCC. In this study, we present baseline (first assessment) and follow-up (second assessment) data from the 130 non-hospitalised participants who completed clinical assessments at two time points. Data were collected between August 2020 and December 2024. The participant selection process is illustrated in a flowchart in Fig. 1.

### Data collection

Data on physical function, physical activity, mental health, and self-rated health were collected from clinical assessments conducted by physiotherapists and psychologists at the post-COVID outpatient clinic. Some self‑reported measures (PHQ‑9, GAD‑7, and EQ‑5D) were collected digitally in connection with the clinical visit, in accordance with prevailing clinical routines. Specific criteria were applied to define impairments and limitations in physical function, physical activity, and mental health, as detailed below [16–21]. These criteria were based on previously established cut-offs where available, or otherwise on thresholds deemed clinically relevant for the purposes of this study, as specified for each outcome below.

*Demographic data* were collected from medical records and included information on sex, age, body mass index (BMI), education, sick leave, smoking status, previous comorbidities, time since COVID-19, a diagnosis of postural orthostatic tachycardia syndrome (POTS) related to COVID-19, Post-COVID-19 functional status scale (PCFS), self-reported symptoms and lung function assessed by dynamic spirometry.

Assessments of *physical function* included the six-minute walk test (6MWT), the one-minute sit-to-stand test (1-min STS) and measurement of inspiratory muscle strength using the maximal inspiratory pressure test (MIP). All tests were performed according to guidelines and are presented as a percentage of the predicted value [16–18, 22–24]. A 6MWT result below the lower limit of normal for the predicted distance was considered indicative of impaired physical function, based on established cut-offs [16]. For both the 1-min STS and MIP, values below the 2.5th percentile of the predicted value were applied to define impairment [17, 18]. Additionally, dyspnoea was assessed using the Modified Medical Research Council dyspnoea scale (mMRC), ranging from 0 to 4. A score of 2 or higher was considered indicative of clinically relevant dyspnoea, based on established cut-offs [21, 25].

Self-reported *physical activity* was assessed using the Frändin/Grimby activity scale (Frändin/Grimby). The scale ranges from 1 (“hardly any physical activity”) to 6 (“hard or very hard physical activity regularly and several times a week”) [26]. In this study, a score below 3 was applied to define limitations in, or low levels of, physical activity.

*Mental health* was assessed by measuring symptoms of depression and anxiety using the Patient Health Questionnaire-9 (PHQ-9) and the Generalised Anxiety Disorder 7-item scale (GAD-7) [18, 19]. The PHQ-9 score ranges from 0 to 27, and the GAD-7 score from 0 to 21. Higher scores indicate more severe symptoms of depression and anxiety. A score of 10 or above on either scale was considered indicative of moderate to severe symptoms of depression or anxiety, based on established cut-offs [19, 20].

*Self-rated health* was measured using the EuroQol visual analogue scale (EQ VAS), included in the EuroQol five-dimension scale **(**EQ-5D) [27]. The EQ VAS is a vertical scale on which individuals rate their current health from 0 (“the worst health you can imagine”) to 100 (“the best health you can imagine”).

### Data analyses

Data were described using mean and standard deviation (SD), median and interquartile ranges (IQR), or frequency (*n*) and proportion (%). For the first aim, impairments and limitations in the outcome variables were described at both assessments. Differences between baseline and follow-up assessments were evaluated using paired t-tests, Wilcoxon signed-rank tests, or McNemar’s tests, depending on data level and distribution after assessment of normality (Shapiro–Wilk). For the second aim, a multiple linear regression analysis was conducted to examine associations between baseline assessment factors and self-rated health at follow-up, using EQ VAS as the dependent variable. As higher values on the EQ VAS indicate better self-rated health, negative associations reflect risk factors for low self-rated health. Independent variables included dichotomised baseline values indicating impairment, defined as 6MWT below the lower limit of normal, 1-min STS and MIP below the 2.5th percentile, mMRC ≥ 2, Frändin/Grimby < 3, PHQ-9 ≥ 10, and GAD-7 ≥ 10. The multivariable model was adjusted for POTS diagnosis, time since COVID-19, age, and sex. These variables were selected based on their clinical relevance in the population and previously reported associations with the outcome variable. All variables were included in the multivariable model simultaneously, ensuring that each variable served as a control for the others, providing a comprehensive analysis of their individual associations with the dependent variable. Assumption checks were done numerically and visually. The level of significance was set at *p* < .05 for all analyses.

Missing data were assumed to be missing at random, as observations with and without missing data on EQ VAS did not differ significantly in the other outcomes. Missing data were imputed using the ‘missForest’ package in R prior to conducting the regression analysis, with up to 40 iterations, resulting in the lowest imputation error (Normalised Root Mean Squared Error = 0.036, Proportion of Falsely Classified = 0.109) [28]. The ‘missForest’ package has been shown to produce low imputation errors for continuous and categorical variables. It employs an iterative imputation scheme, fitting a random forest model for each variable on the remaining imputed dataset. The model is trained on observed data and applied to missing data, starting with the variable with the least missing data. Predictions from the trained model are then used to update the imputed dataset.

To assess potential selection bias related to loss to follow‑up, a sensitivity analysis was conducted comparing participants included in the analytic sample, who completed both the baseline and follow‑up assessments, with those who completed only one physical assessment. Demographic variables and baseline outcomes were compared using Welch’s t‑tests, Mann–Whitney U tests, and chi‑square or Fisher’s exact tests, depending on the level and distribution of the data.

## Results

### Participants

**Fig. 2:**
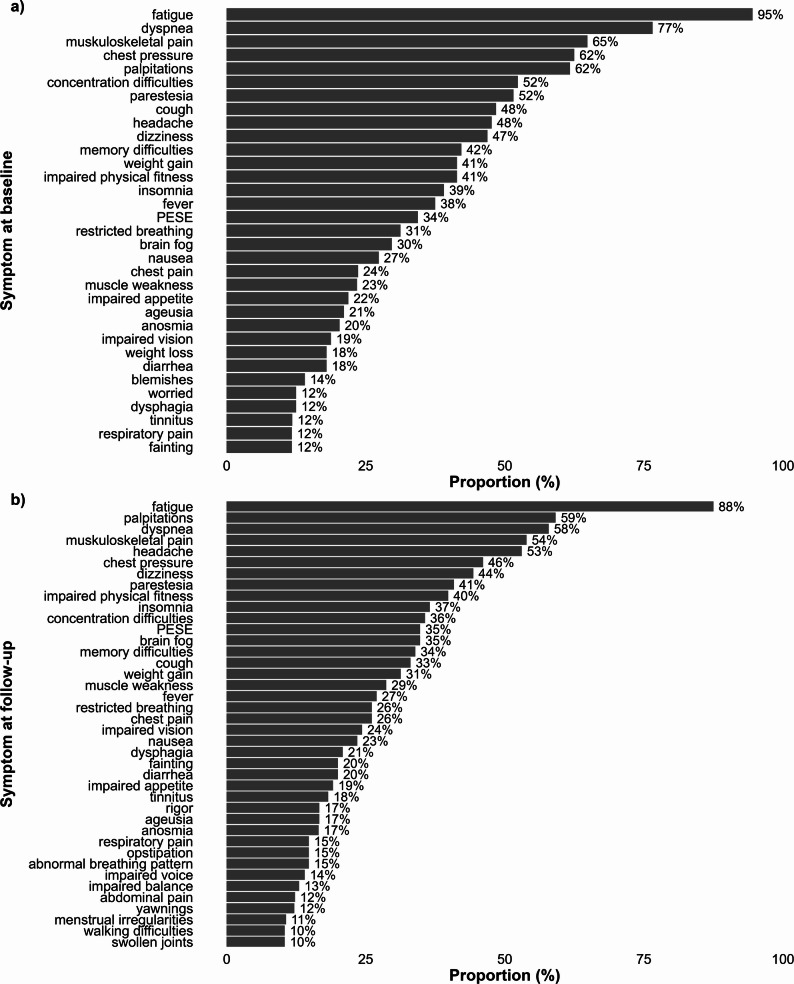
The most common self-reported symptoms (≥10%) at **a**) the baseline assessment at in mean 12 months after COVID-19 (*n* = 128) and **b**) at the follow-up assessment at in mean 30 months after COVID-19

Demographic and clinical characteristics of the 130 included participants are presented in Table 1. The study sample mainly consisted of middle-aged women, who prior to COVID-19 were working or studying, had a high educational level, reported high levels of physical activity, and had no previous functional limitations. The most common previous co-morbidities were asthma (16%), hypertension (12%), a history of burnout (11%), and depression (10%). All self-reported symptoms at both assessments are presented in Fig. 2. The most commonly reported symptoms at both time points were fatigue, dyspnoea, musculoskeletal pain, chest pressure, headache, and palpitations.

**Table 1:** Demographics and clinical characteristics of participants in the study sample (*n* = 130)

Characteristics	Value
Sex (female)	113 (87%)
Age (yrs)	46 (10)
BMI (kg/m^2^)	25.3 (5.7)
Higher education (> 13 yrs)	106 (95%)^19^
Sick leave ≥ 50%	
* Before COVID-19*	5 ( 4%)^3^
* Baseline assessment*	91 (74%)^7^
* Follow-up assessment*	69 (53%)
Never smoker	94 (72%)
Previous co-morbidities	
* Number of co-morbidities*	1 (1–2)^1^
* Asthma*	21 (16.3%)^1^
* Hypertension*	16 (12.4%)^1^
* Burnout*	14 (10.8%)^1^
* Depression*	13 (10.0%)^1^
* Cancer or tumour disease*	8 (6.2%)^1^
* Anxiety*	7 (5.4%)^1^
* Diabetes mellitus*	3 (2.3%)^1^
* Rheumatological disease*	3 (2.3%)^1^
* Cardiac arrhythmia*	2 (1.6%)^1^
* Thrombosis or pulmonary embolism*	2 (1.6%)^1^
* Chronic obstructive pulmonary disease*	0 (0%)^1^
* Pulmonary fibrosis*	0 (0%)^1^
* Neurological disease*	0 (0%)^1^
* Renal disease*	0 (0%)^1^
* Other cardiac conditions (angina*,* MI*,* heart failure)*	0 (0%)^1^
POTS diagnosis after COVID-19	43 (33%)
Months since COVID-19	
* Baseline assessment*	12 (5)
* Follow-up assessment*	30 (10)
Physical activity before COVID-19 (Frändin/Grimby)	5 (4–5)
Post-COVID-19 functional status	
* Before COVID-19*	0 (0–0)^6^
* Baseline assessment*	3 (2–3)^4^
* Follow-up assessment*	3 (2–3)^12^
Number of symptoms, at assessments	
* Baseline assessment*	13 (10–17)^2^
* Follow-up assessment*	15 (7–16)^14^
Lung function, at baseline assessment	
* FEV*_*1*_ *(% of predicted)*	88 (13)^20^
* FVC (% of predicted)*	88 (13)^20^

Values are presented as mean (SD), median (IQR), or *n* (%), with missing data indicated in superscript where applicable. *Abbreviations*: *BMI* body mass index, *FEV*_1_ forced expiratory volume in one second, *Frändin/Grimby* Frändin/Grimby activity scale, *FVC* forced vital capacity, *MI* myocardial infarction, *POTS* Postural orthostatic tachycardia syndrome

### Impact on physical function, physical activity, mental health, and self-rated health

Table 2 presents data on physical function, physical activity, mental health, and self-rated health at baseline and follow-up, as well as the changes observed between the two time points. Significant improvements were observed across all outcome measures, with the exception of 1-min STS and GAD-7, which showed no significant change.

**Table 2:** Outcomes in physical function, physical activity, mental health and self-rated health at the baseline and follow-up, including changes observed between the two assessments

Outcome	Baseline	Follow-up	Diff [95% CI]^a^	*p*-value
Physical function				
6MWT (% pred)	80 (26)^3^	85 (25)^3^	6 [3 to 9]^6^	<.001*
1-min STS (% pred)	67 (29)^5^	67 (28)^14^	0 [-4 to 5]^19^	.822
MIP (% pred)	87 (31)	98 (28)^1^	12 [9 to 15]^1^	<.001*
mMRC dyspnoea	2 (2-3)^10^	2 (1-3)^37^	-1 [-1 to -1]^43^	<.001*
Physical activity				
Frändin/Grimby	2 (2-3)^1^	3 (2-3)^7^	1 [1 to 1]^8^	<.001*
Mental health				
PHQ-9	11 (7-15)^5^	10 (5-14)^35^	-2 [-3 to 1]^37^	<.001*
GAD-7	5 (2-9)^4^	4 (2-9)^34^	-1 [-2 to 0]^36^	.072
Self-rated health				
EQ VAS^b^	38 (19)^6^	47 (23)^47^	8 [3 to 13]^49^	<.001*

Data are presented as mean (SD), median (IQR), and mean difference with 95% confidence intervals [95% CI]. Missing values are reported in superscript after each value where applicable. Statistical significance is indicated by* p* < .05 (*)

*Abbreviations*: *1-min STS* 1 minute sit-to-stand test, *6MWT* six-minute walk test, *EQ VAS* EQ-5D visual analogue scale, *Frändin/Grimby* Frändin/Grimby activity scale, *GAD-7* General Anxiety Disorder 7-item scale, *MIP* maximal inspiratory pressure, *mMRC dyspnoea* Modified Medical Research Council Dyspnea Scale, *PHQ-9* Patient Health Questionnaire-9

^a^Mean differences are based on paired t‑tests; Hodges–Lehmann estimates are used for Wilcoxon signed‑rank tests

^b^Mean (SD) for imputed follow-up data: 47 (19)

**Fig. 3:**
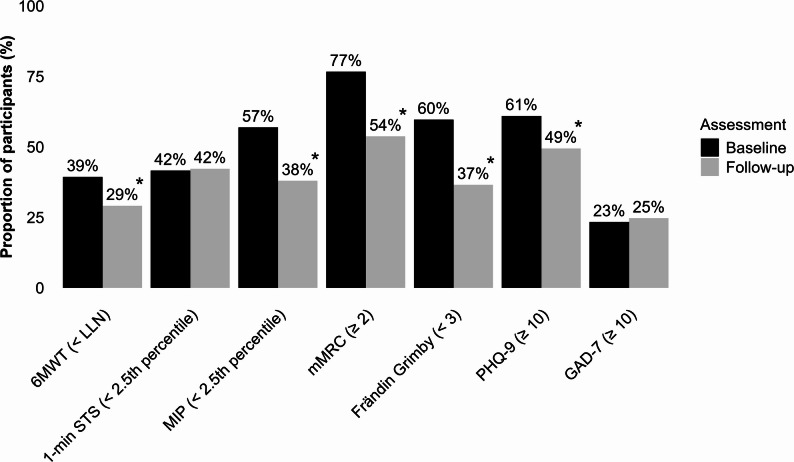
Proportions of participants with impairments and limitations in physical function, physical activity, and mental health at baseline and follow-up, based on predefined criteria. Outcomes demonstrating statistically significant changes between the two assessments are indicated by an asterisk (*). Abbreviations: 1-min STS: 1-minute sit-to-stand test; 6MWT: Six-minute Walk Test; Frändin/Grimby: Frändin/Grimby activity score; GAD-7: General Anxiety Disorder 7-item scale; MIP: maximal inspiratory pressure; mMRC: Modified Medical Research Council Dyspnea Scale; PHQ-9: Patient Health Questionnaire-9

Figure 3 illustrates the proportions of participants reporting impairments or limitations in each outcome measure at both time points, as well as observed changes between assessments. The proportion of participants with impairments and limitations decreased significantly for all outcomes, except for the 1-min STS and GAD-7, which remained unchanged. At follow-up, impairments were most frequently observed in mMRC (54%), PHQ-9 (49%), and the 1-min STS (42%) (Fig. 3). 

### Risk factors for lower self-rated health

**Fig. 4:**
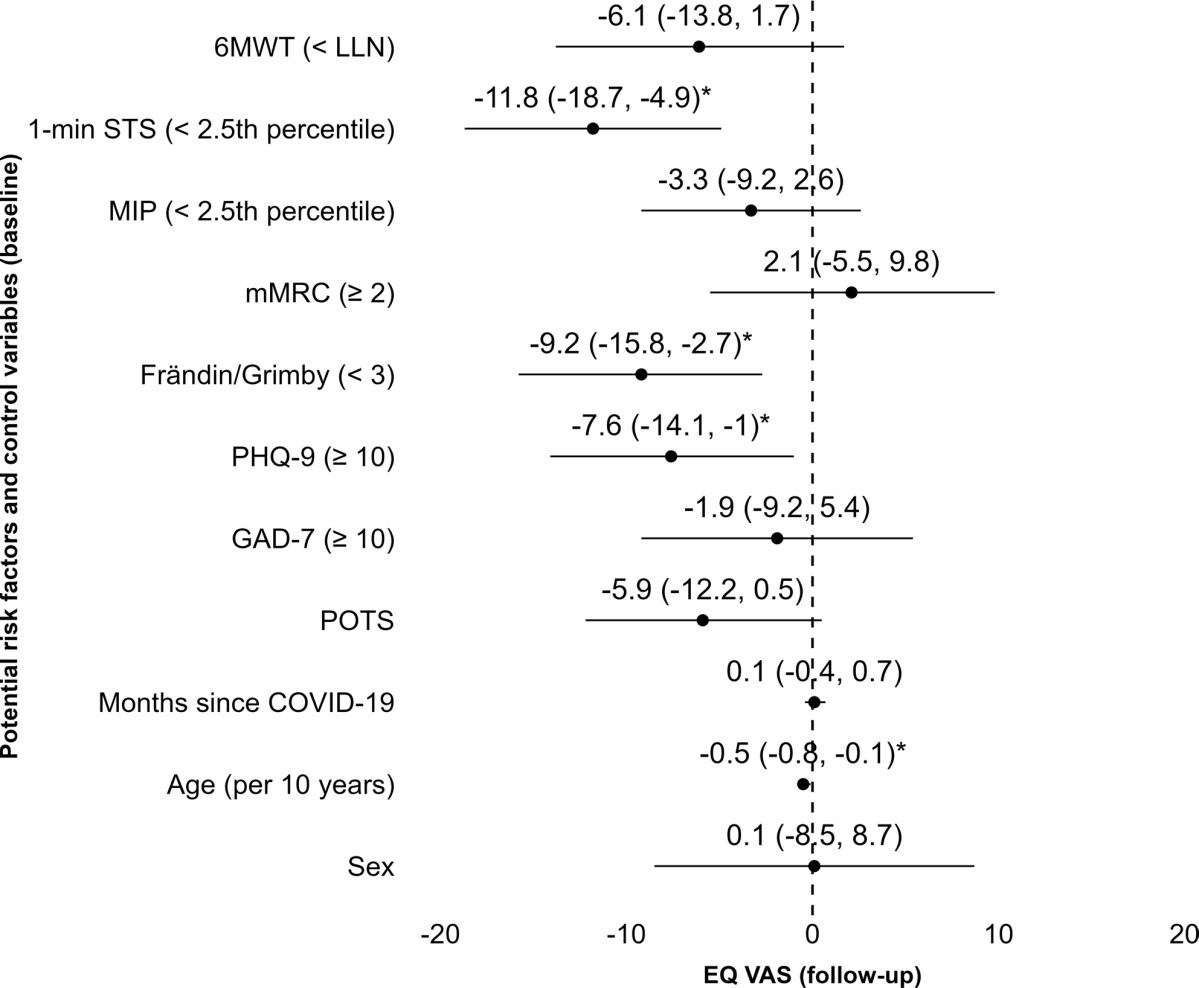
Forest plot illustrating the associations of potential baseline risk factors (at 12 months) and control variables with EQ VAS scores at follow-up assessment (at 30 months), based on a multivariable linear regression model. Unstandardised regression coefficients (b) are presented with 95% confidence intervals. Statistically significant associations (*p* < .05) are indicated by an asterisk (*). Multivariable model: F (11,118) = 5.918, *n* = 130, R2 = 0.36, R2-adj = 0.30, *p* < .001. All VIF: < 2. Intercept: 90.7 (95% CI: 76.2; 110). Abbreviations: 1-min STS: 1-minute sit-to-stand test; 6MWT: Six-minute Walk Test; Frändin/Grimby: Frändin/Grimby activity score; GAD-7: General Anxiety Disorder 7-item scale; MIP: maximal inspiratory pressure; mMRC: Modified Medical Research Council Dyspnea Scale; PHQ-9: Patient Health Questionnaire-9

In the multivariable linear regression model, impaired performance in the 1-min STS (< 2.5th percentile), a low physical activity level (Frändin/Grimby < 3), and depressive symptoms (PHQ-9 score ≥ 10) at baseline were all significantly associated with lower EQ VAS scores at follow-up, after adjusting for POTS diagnosis, time since COVID-19, age, and sex, as illustrated in a forest plot in Fig. 4. Although age was included as a covariate, older age was independently associated with lower EQ VAS scores. 

In the sensitivity analysis, participants included in the analytic sample (n = 130) did not differ significantly from those with only one physical assessment (n = 337) on most demographic or baseline outcome variables. The analytic sample had slightly higher sick leave at baseline (74% vs 59%, p = .004), reported more symptoms (13 [10–17] vs 12 [9–14], p = .009), showed higher dyspnoea scores (mMRC: 2 [2–3] vs 2 [1–3], p < .001), and had lower self‑reported physical activity on the Frändin/Grimby scale (2 [2–3] vs 3 [2–3], p = .012) compared with the single‑visit group. A table summarising these comparisons is provided in Supplementary Table S1.

## Discussion

In this cohort study, we observed sustained negative impacts of PCC on health outcomes 2.5 years after initial COVID-19 in non-hospitalised individuals, despite minor improvements over time. Prevalent issues included impairments in physical function and mental health, low physical activity levels, and reduced self-rated health. At 2.5 years, approximately half of the participants demonstrated impaired performance in the 1-min STS, severe dyspnoea, and symptoms of moderate to severe depression, underscoring the persistent burden on both physical and mental health and the long-term rehabilitation needs. Risk factors for low self-rated health at 2.5 years included impaired physical function (1-min STS < 2.5th percentile), low physical activity level (Frändin/Grimby < 3), and depressive symptoms (PHQ-9 ≥ 10) assessed at 12 months.

Although most outcomes showed statistically significant improvements between assessments, the changes may not represent clinically meaningful improvement. These findings contribute to the understanding of the clinical course of PCC, suggest a slow improvement over time, and highlight the need for further research to better characterise recovery trajectories in this population.

Our findings of improved but persisting impairments in physical function at 2.5 years extend previous research by showing that such impact can remain beyond a year in non-hospitalised individuals with PCC, a group for whom long-term objective assessments are scarce [6, 12]. Our baseline assessment, conducted at 12 months post-infection, broadly aligns with previous findings on non-hospitalised individuals with PCC, showing similar impairment in physical function (6MWT, 1-min STS, mMRC, MIP) up to one year following COVID-19 [6, 12]. When comparing our findings to previously reported long-term outcomes in hospitalised individuals, we observed greater impairments in physical function among our non-hospitalised cohort [6, 8, 9]. Specifically, a recent meta-analysis reported a mean of 93% of predicted distance in the 6MWT at ≥ 11 months post-COVID-19 in both hospitalised and non-hospitalised individuals, compared to 85% at 2.5 years in our study [6]. Furthermore, a follow-up study of hospitalised individuals found that 12% reported dyspnoea three years after discharge, whereas 54% of participants in our study had a mMRC ≥ 2 at follow-up [9]. These discrepancies may be attributed to differences in study populations. Our cohort consisted exclusively of non-hospitalised individuals with severe PCC referred to a specialised clinic, whereas previous studies may have included participants with a broader range of PCC severity [6, 8, 9].

Given that most participants self-reported high physical activity levels prior to COVID-19, our finding of persistently low activity levels several years after COVID-19 is noteworthy. This decline is consistent with previous research based on self-reported physical activity up to one year after COVID-19 onset [29, 30]. A plausible explanation is that symptoms and impairments associated with PCC may limit individuals’ ability to resume or maintain previous activity levels over time. Supporting this interpretation, studies using objective methods such as activity monitors—including a study by Rosa‑Souza et al. and recently published work within the ReCOV project—have demonstrated associations between PCC‑related symptoms or impairments, and lower levels of physical [31, 32].

The prevalence of symptoms indicating moderate to severe depression and anxiety was similar to that reported in previous studies of non-hospitalised individuals but appeared higher compared to findings in hospitalised populations [7, 8]. The pre-COVID-19 prevalence of depression in our sample was 10%, which does not fully account for the substantially higher proportion of participants scoring ≥ 10 on the PHQ-9 (baseline: 61%; follow-up: 49%), indicating moderate to severe depressive symptoms [19]. This elevated prevalence may, at least in part, be attributable to the condition itself.

Another important finding was the markedly poor self-rated health, with EQ VAS scores substantially lower than the Swedish population mean of 76 (SD 19) at both time points (baseline: 38 [SD 19]; follow-up: 47 [SD 23]) [33]. EQ VAS scores in our cohort were also generally lower than those previously reported in individuals with PCC [7, 8]. A 2024 meta-analysis reported only a small reduction in self-rated health compared to healthy controls, with EQ VAS scores ranging from 66 to 87 [7]. This difference may be explained by the greater observed physical and mental impairments, as well as the higher severity of PCC in our study population.

The identified risk factors for lower self-rated **–** including impaired physical performance (1-min STS < 2.5th percentile), low physical activity level (Frändin/Grimby < 3), and depressive symptoms (PHQ-9 ≥ 10) – underscore the importance of identifying individuals with physical and psychological vulnerability to improve long-term health outcomes. The clinical relevance of these findings is supported by the magnitude of the observed associations. Estimated differences in EQ VAS scores were 8–10 points lower for those with impaired physical performance, low activity levels or depressive symptoms – exceeding the suggested minimal clinically important difference (MCID) of 7.5 points for this population [34]. However, the confidence intervals were relatively wide, indicating uncertainty in the precise estimates and warranting cautious interpretation. These results align with previous findings linking poor mental health and low physical activity to lower self-rated health in non-hospitalised individuals with PCC [34]. Clinically, these findings support systematic follow-up using simple tools such as the 1-min STS, Frändin/Grimby scale, and PHQ-9 – enabling earlier, targeted interventions to improve long-term outcomes and reduce the burden of persistent symptoms and long-term sequelae.

### Methodological considerations

Although the study design allows for temporal ordering, the identified risk factors reflect statistical associations rather than confirmed causal relationships. The representativeness of our sample warrants consideration, as only individuals with severe PCC, recruited from a specialised outpatient clinic, were included. Approximately half of the eligible patients participated in the ReCOV project. Variations in recruitment procedures, logistics, and clinical routines meant that some individuals were not approached, which may have introduced selection bias and limited generalisability. Moreover, the relatively small sample size may further limit the generalisability of the findings, particularly to individuals with milder forms of the condition. The sample predominantly consisted of middle‑aged women with high pre‑COVID physical activity levels and no previous functional limitations. While these characteristics may influence recovery and limit generalisability, they reflect patterns commonly observed in PCC populations [3, 29, 30].

The sensitivity analysis further indicates potential selection bias, as participants who completed both assessments exhibited somewhat higher baseline symptom burden and lower physical activity compared with those who only attended one visit. Individuals with more pronounced symptoms may have been more inclined to return for follow‑up, whereas those with milder symptoms may have been less likely to do so, potentially influencing estimates of recovery.

The study did not include a healthy control group; instead, results were compared with established reference values and clinically relevant cut-offs. However, participants were followed longitudinally and thus served as their own controls. It is possible that improvements were underestimated, as individuals who had fully recovered may not have returned for follow-up and were therefore not included in the analysis.

Furthermore, changes in clinical routines over time meant that not all physical tests were performed at every assessment, and because several self-reported measures were collected digitally, some participants attended their appointments but did not complete the electronic questionnaires, resulting in increased missing data for certain outcomes. Missing data were addressed using a robust imputation method, which enabled the inclusion of a larger sample and helped reduce potential bias associated with complete case analysis. Data collection during an ongoing pandemic led to varying follow-up times, which was accounted for in the regression model. The model was further adjusted for age, sex, and the presence of a POTS diagnosis to address potential confounding. Despite these limitations, the study included a comprehensive battery of validated assessments and had a relatively low overall proportion of missing data, strengthening the reliability of the findings.

## Conclusion

This study highlights the long-term negative health impacts of PCC in previously non-hospitalised adults, including persistent impairments in physical and mental health, low physical activity, and low self-rated health up to 2.5 years after COVID-19. Impaired physical function, low activity levels, and depressive symptoms were identified as risk factors for low self-rated health. These findings contribute to a deeper understanding of the long-term trajectory of PCC and support identification of individuals at risk of poor health outcomes through systematic follow-up. The use of simple, clinically applicable tools may facilitate detection and enable targeted rehabilitation strategies in this population.

## Supplementary Information

Supplementary Material 1.

## Data Availability

Data is not publicly available, but available upon request. Requests for access to the data can be put to our Research Data Office (rdo@ki.se) at Karolinska Institutet and will be handled according to the relevant legislation. This will require a data processing agreement or similar with the recipient of the data.

## Abbreviations

1-min STS: 1-minute sit-to-stand test
6MWT: Six-minute Walk Test
BMI: body mass index
EQ-5D: EuroQol five-dimension scale
EQ VAS: EuroQol visual analogue scale
FEV_1_: forced expiratory volume in one second
Frändin/Grimby: Frändin/Grimby activity score
FVC: forced vital capacity
GAD-7: General Anxiety Disorder 7-item scale
IQR: interquartile range; MIP: maximal inspiratory pressure
mMRC: Modified Medical Research Council Dyspnea Scale
PHQ-9: Patient Health Questionnaire-9
PCC: post-COVID-19 condition
POTS: Postural orthostatic tachycardia syndrome
ReCOV: Recovery and rehabilitation during and after COVID-19
SD: standard deviation
