# Supplementary material for: Long-term health outcomes and risk factors for low self-rated health in non-hospitalised adults with post-COVID-19 condition: a 2.5-year cohort study
Source: BMC Public Health. 2026 Feb 3;26:715. doi: 10.1186/s12889-026-26532-z (PMC12930953; doi:10.1186/s12889-026-26532-z)
Supplement: Supplementary file 1 — Supplementary Material 1. [file 12889_2026_26532_MOESM1_ESM.docx]

| **Table S1.** Sensitivity analysis comparing demographic and clinical outcome variables at baseline assessment between the analytic sample (n = 130) and the single visit group (n = 337). | | | | | |
| --- | --- | --- | --- | --- | --- |
| **Characteristics** | | | **Analytic sample**  (n = 130) | **Single visit group**  (n = 337) | **Diff**  (p value) |
| Sex (female) | | | 113 (87%) | 279 (83%) | .342 |
| Age (yrs) | | | 46 (10) | 45 (12) | .382 |
| BMI (kg/m^2^) | | | 25.3 (5.7) | 25.5 (4.8) | .729 |
| Higher education (> 13 yrs) | | | 106 (95%)^19^ | 243 (91%)^71^ | .375 |
| Sick leave ≥ 50% | | |  |  |  |
|  | *Before COVID-19* | | 5 ( 4%)^3^ | 11 (4%) | .809 |
|  | *Baseline assessment* | | 91 (74%)^7^ | 188 (59%)^20^ | .004* |
| Never smoker | | | 94 (72%) | 239 (71%) | .766 |
| Previous comorbidities | | |  |  |  |
|  | | *Number of co-morbidities* | 1 (1-2) | 1 (0-2) | .261 |
|  | | *Asthma* | 21 (16.3%)^1^ | 54 (16.2%)^4^ | .987 |
|  | | *Hypertension* | 16 (12.4%)^1^ | 37 (11.1%)^4^ | .153 |
|  | | *Burnout* | 14 (10.8%)^1^ | 51 (15.1%)^4^ | .222 |
|  | | *Depression* | 13 (10.0%)^1^ | 34 (10.1%)^4^ | .977 |
|  | | *Cancer or tumour disease* | 8 (6.2%)^1^ | 14 (4.3%)^4^ | .371 |
|  | | *Anxiety* | 7 (5.4%)^1^ | 19 (5.6%)^4^ | .915 |
|  | | *Diabetes mellitus* | 3 (2.3%)^1^ | 9 (3.7%) | 1.00 |
|  | | *Rheumatological disease* | 3 (2.3%)^1^ | 11 (3.3%)^4^ | .766 |
|  | | *Cardiac arrhythmia* | 2 (1.6%)^1^ | 14 (4.3%)^4^ | .255 |
|  | | *Thrombosis or pulmonary embolism* | 2 (1.6%)^1^ | 0 (0%)^4^ | .078 |
|  | | *Chronic obstructive pulmonary disease* | 0 (0%)^1^ | 1 (0.5%)^4^ | 1.00 |
|  | | *Pulmonary fibrosis* | 0 (0%)^1^ | 0 (0%)^4^ | - |
|  | | *Neurological disease* | 0 (0%)^1^ | 5 (1.5%)^4^ | .329 |
|  | | *Renal disease* | 0 (0%)^1^ | 3 (0.9%) | .563 |
|  | | *Other cardiac conditions (angina, MI, heart failure)* | 0 (0%)^1^ | 0 (0%)^4^ | - |
| POTS diagnosis after COVID-19 | | | 43 (33%) | 99 (29%) | .395 |
| Months since COVID-19 | | | 12 (5) | 16 (15) | >.001* |
| Physical activity (Frändin/Grimby) | | |  |  |  |
|  | | *Before COVID-19* | 5 (4-5) | 5 (4-6) | .283 |
|  | | *Baseline assessment* | 2 (2-3)^1^ | 3 (2-3)^6^ | .012* |
| Post-COVID-19 functional status | | |  |  |  |
|  | *Before COVID-19* | | 0 (0-0) | 0 (0-0)^22^ | .711 |
|  | *Baseline assessment* | | 3 (2-3)^4^ | 3 (2-3)^14^ | .257 |
| Number of symptoms, at baseline assessment | | | 13 (10-17)^2^ | 12 (9-14)^3^ | .009* |
| Lung function, at baseline assessment | | |  |  |  |
|  | *FEV_1_ (% of predicted)* | | 88 (13)^20^ | 88 (14)^35^ | .850 |
|  | *FVC (% of predicted)* | | 88 (13)^20^ | 88 (13)^35^ | .975 |
| Physical function, at baseline assessment | | |  |  |  |
|  | *6MWT (% pred)* | | 80 (26)^3^ | 80 (22)^6^ | .893 |
|  | *1-min STS (% pred)* | | 67 (29)^5^ | 66 (26)^26^ | .881 |
|  | *MIP (% pred)* | | 87 (31) | 87 (26)^5^ | .944 |
|  | *mMRC dyspnoea* | | 2 (2-3)^10^ | 2 (1-3)^23^ | <.001* |
| Mental health, at baseline assessment | | |  |  |  |
|  | *PHQ-9* | | 11 (7-15)^5^ | 11 (6-15)^23^ | .360 |
|  | *GAD-7* | | 5 (2-9)^4^ | 5 (2-9)^19^ | .833 |
| Self-rated health, at baseline assessment | | |  |  |  |
|  | *EQ VAS* | | 38 (19)^6^ | 41 (18)^22^ | .156 |
| Values are presented as mean (SD), median (IQR), or n (%), with missing data indicated in superscript where applicable. Group comparisons were performed using Welch’s t‑test for continuous variables, the Mann–Whitney U test for ordinal variables, and chi‑square or Fisher’s exact tests for categorical variables depending on expected cell counts. Statistical significance is indicated by p < .05 (*).  Abbreviations: 1‑min STS: 1‑minute sit‑to‑stand test; 6MWT: six‑minute walk test; BMI: body mass index; EQ VAS: EQ‑5D visual analogue scale; FEV_1_: forced expiratory volume in one second; FVC: forced vital capacity; Frändin/Grimby: Frändin/Grimby activity scale; GAD‑7: Generalized Anxiety Disorder‑7; MI: myocardial infarction; mMRC dyspnoea: Modified Medical Research Council Dyspnea Scale; MIP: maximal inspiratory pressure; PHQ‑9: Patient Health Questionnaire‑9; POTS: postural orthostatic tachycardia syndrome. | | | | | |
